# Quantum chemical investigation and molecular design of coumarin-based heavy-metal-free photosensitizers for one- and two-photon excited fluorescence imaging and photodynamic therapy

**DOI:** 10.1039/d5ra07339a

**Published:** 2026-01-19

**Authors:** Thanh Chung Pham, Dung Ngoc Tran, Van Trang Nguyen, Van Thong Pham, Dai Lam Tran, Songyi Lee

**Affiliations:** a Institute of Materials Science, Vietnam Academy of Science and Technology 18 Hoang Quoc Viet, Cau Giay Hanoi Vietnam ptchung@ims.vast.vn; b Faculty of Chemistry, Hanoi National University of Education Hanoi Vietnam; c R&D Center, Vietnam Education and Technology Transfer JSC Cau Giay Hanoi Vietnam; d Industry 4.0 Convergence Bionics Engineering, Pukyong National University Busan 48513 Korea; e Department of Chemistry, Pukyong National University Busan 48513 Korea

## Abstract

The rational design of heavy-metal-free photosensitizers (PSs) is essential for advancing fluorescence (FL) imaging and photodynamic therapy (PDT). In this work, we present a systematic quantum-chemical investigation of eight coumarin-based derivatives (C1–C8) to elucidate how molecular structure controls excited-state dynamics. Time-dependent density functional theory (TD-DFT), combined with Fermi's Golden Rule, was applied to compute FL emission, internal conversion (IC), and intersystem crossing (ISC) rate constants, enabling quantitative prediction of FL and triplet quantum yields. The results show that C1, C2 and C6 undergo reduced fluorescence due to partial population of the dark ^1^TICT state, but maintains both moderate fluorescence and appreciable triplet yield, supporting dual applications in imaging and PDT. The heavy-atom derivative C3 achieves nearly unit triplet quantum yield (*Φ*_T_ ≈ 1.0), confirming the dominant role of sulfur-enhanced ISC and reactive oxygen species generation. In contrast, C4 and C8 favor fluorescence over ISC, while C5 and C7 exhibit the highest emission efficiency by suppressing both TICT state and ISC process, identifying them as optimal imaging probes. Importantly, Herzberg–Teller vibronic coupling was found to dominate ISC efficiency in heavy-atom-free systems but was negligible in heavy-atom-based analogues. In addition, the large two-photon absorption (TPA) cross sections of C1–C8 provide redshifted excitation windows, thereby overcoming the penetration limitations of one-photon absorption (OPA) and enhancing biomedical applicability. Collectively, these insights establish design principles for tailoring radiative and non-radiative pathways in coumarin scaffolds, enabling the targeted development of multifunctional organic PSs.

## Abstract 2

The rational design of heavy-metal-free photosensitizers (PSs) is essential for advancing fluorescence (FL) imaging and photodynamic therapy (PDT).

## Graphical abstract

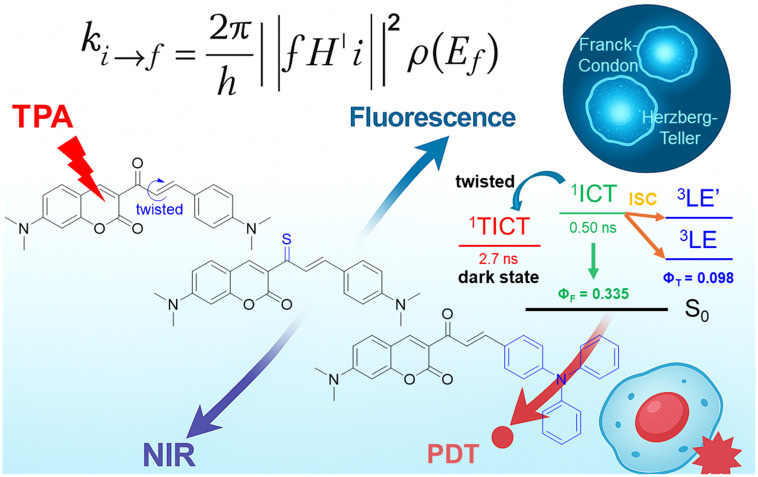

## Introduction

Photodynamic therapy (PDT) has established itself as a minimally invasive and highly selective therapeutic modality for cancer treatment and antimicrobial applications.^1,2^ By relying on light-triggered activation of photosensitizers (PSs) to generate reactive oxygen species (ROS), PDT achieves localized cytotoxicity with reduced side effects compared with conventional approaches such as chemotherapy and radiotherapy. Its capacity for spatial and temporal control, repeatability, and immune-system stimulation has driven increasing clinical and research interest.^3–6^

The efficacy of PDT is fundamentally determined by the photophysical properties of the PS.^7^ Conventional PSs often incorporate heavy atoms, including transition metals, iodine, or bromine, to accelerate intersystem crossing (ISC) and enhance ROS generation.^8,9^ However, this strategy is accompanied by significant drawbacks such as dark cytotoxicity, high cost, and poor biocompatibility.^9^ In response, recent research has focused on heavy-metal-free PSs, which promise safer and more sustainable alternatives. Yet, without heavy atoms, ISC becomes less efficient, compromising ROS production.^10–12^ Moreover, the interplay between radiative decay, nonradiative internal conversion, and ISC often leads to fluorescence quenching, limiting the dual application of such PSs in both therapy and bioimaging.^13^

Mechanistic studies have revealed that the efficiency of heavy-atom-free PSs is strongly influenced by molecular structure. Spin–orbit coupling (SOC),^14–17^ singlet–triplet energy gaps (Δ*E*_ST_),^18–20^ and the formation of dark twisted intramolecular charge-transfer (^1^TICT) states^21–24^ are critical parameters governing the balance between fluorescence and ROS generation. Strategies such as donor–acceptor engineering,^18,19^ π-conjugation extension,^25^ and carbonyl-to-thiocarbonyl substitution^14,16,17^ have been proposed to modulate these factors. Despite promising advances, a detailed quantum-chemical framework connecting molecular design to excited-state dynamics remains incomplete. This gap hinders the rational development of multifunctional PSs that can simultaneously provide strong fluorescence signals for imaging and efficient triplet formation for PDT.

π-Conjugated organic chromophores are attractive for photonic/biophotonic applications because their structures can be tuned to control both linear (OPA absorption/emission and fluorescence efficiency) and nonlinear optical responses (*e.g.*, TPA cross sections). In donor–acceptor systems, these responses are closely linked to intramolecular charge transfer (ICT), *i.e.*, excitation-induced electron–hole redistribution that governs spectral positions, transition strengths, and relaxation pathways. Importantly, ICT is regulated not only by donor/acceptor strength and π-conjugation, but also by molecular topology/branching and microenvironmental effects such as solvent polarity and local rigidity, which together determine whether the excited state evolves into a stabilized CT/TICT state and how efficiently two-photon excitation is expressed.^26^ This ICT-driven tunability also underlies a key challenge for heavy-metal-free multifunctional PSs: while enhanced CT character can redshift absorption and strengthen nonlinear response, excessive charge separation and conformational relaxation can reduce electron–hole overlap, increase nonradiative decay *via* dark CT/TICT states, and thereby compromise emissive output and/or productive photochemistry; recent experimental and theoretical studies further support this structure-ICT-optical-response relationship and its dependence on topology and environment.^27,28^

Coumarin derivatives represent an attractive platform for this pursuit. Their synthetic accessibility, structural versatility, and inherent photostability enable systematic tuning of electronic and optical properties.^21,29–32^ In particular, coumarins support both one-photon absorption (OPA) and two-photon absorption (TPA), the latter offering advantages for deep-tissue imaging through near-infrared (NIR) excitation.^33–37^

In this study, we present a comprehensive computational investigation of eight coumarin-based derivatives (C1–C8). Using time-dependent density functional theory (TD-DFT), quadratic response theory, and rate calculations based on Fermi's Golden Rule, we analyze how molecular modifications, including thiocarbonyl substitution, π-conjugation extension, and donor–acceptor tuning, govern excited-state dynamics. We quantify fluorescence, internal conversion, and ISC rates, correlate them with experimental data, and evaluate one- and two-photon absorption properties. The insights gained establish structure–property–function relationships that clarify how to suppress nonradiative decay, promote efficient ISC, or balance both pathways. In doing so, this work not only rationalizes the photophysical behavior of coumarin PSs but also defines design principles for next-generation heavy-metal-free sensitizers with dual functionality in fluorescence imaging and photodynamic therapy. Looking ahead, the mechanistic understanding and design strategies outlined here may extend beyond coumarins, providing a general framework for engineering diverse classes of organic chromophores into multifunctional photosensitizers tailored for precision medicine.

## Experimental

### Theoretical method and computational details

The ground-state (S_0_) geometries of the designed PSs were optimized employing the PBE0 hybrid functional^38^ in conjunction with the def2-TZVP basis set,^39^ ensuring the absence of imaginary frequencies. Prior to the mechanistic calculations, we benchmarked TD-DFT absorption/emission energies for C1, C2 and C7 against experiment using representative GGA, hybrid GGA (including PBE0 and PBE38), meta/hybrid *meta*-GGA, and range-separated hybrid functionals (Fig. S1). PBE0 provided the best overall agreement (lowest MAD) for this coumarin ICT/HLCT manifold; therefore, PBE0 was adopted for the production calculations. Solvent effects were incorporated using the conductor-like polarizable continuum model (CPCM)^40^ with toluene. Subsequently, one-photon absorption (OPA) properties were computed at the same theoretical level within the Tamm–Dancoff approximation (TDA)^41^ to TD-DFT, based on the optimized S_0_ geometries. The lowest singlet excited states (S_1_ and 
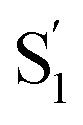
), along with the triplet states (T_1_, T_2_, and T_3_), were optimized using TDA TD-DFT at the PBE0/def2-TZVP level of theory. Additionally, the angle scan of the 
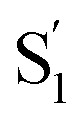
 state was carried out with full TD-DFT at the same computational level. All DFT and TD-DFT calculations were performed using the Gaussian 16 software package.^42^ Hole–electron distribution analyses were performed and visualized using the Multiwfn package.^43^

The two-photon absorption (TPA) properties of the coumarin-based PSs were investigated within the framework of quadratic response theory,^44^ employing the CAM-B3LYP functional^45^ as implemented in the DALTON program package.^46^ TPA cross section (GM) was derived from the two-photon transition probability (*δ*_au_) according to the relation:^47^1
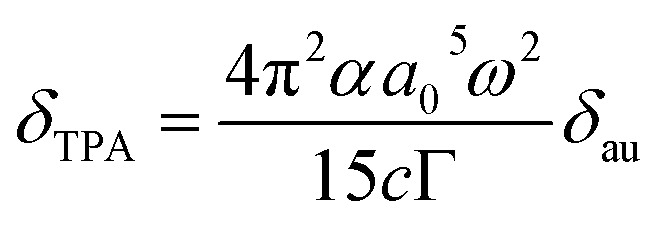
Here, *α* denotes the fine-structure constant, *a*_0_ represents the Bohr radius, *ω* corresponds to the photon energy expressed in atomic units, *c* is the speed of light, and *Γ* is the phenomenological broadening parameter accounting for spectral line broadening associated with electronic excitations.

Fluorescence (FL), internal conversion (IC), and intersystem crossing (ISC) rate constants were computed using the ORCA 6.0 program package.^48,49^ The calculations were carried out within the framework of the adiabatic Hessian model^50,51^ and PBE0/def2-TZVP level of theory, applying Fermi's Golden Rule as formulated in eqn (2):^52–55^2
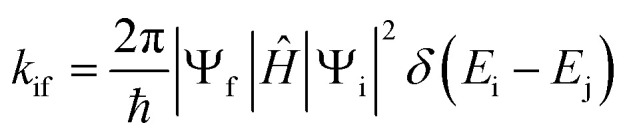
Here, (non-)radiative decay rate (*k*_if_) denotes the transition probability per unit time from an initial state (i) to a final state (f), mediated by a time-independent perturbation Hamiltonian operator (*Ĥ*) and the Dirac delta function (*δ*) enforcing energy conservation. Within the Born-Oppenheimer approximation, the total wavefunctions (*Ψ*) of the initial and final states are factorized into their respective electronic and vibrational components, thereby enabling the separation of electronic and nuclear contributions to the transition matrix elements. Further methodological details and computational protocols are elaborated in the Discussion section.

## Results and discussion

### Molecular design and electronic structure

**Fig. 1:**
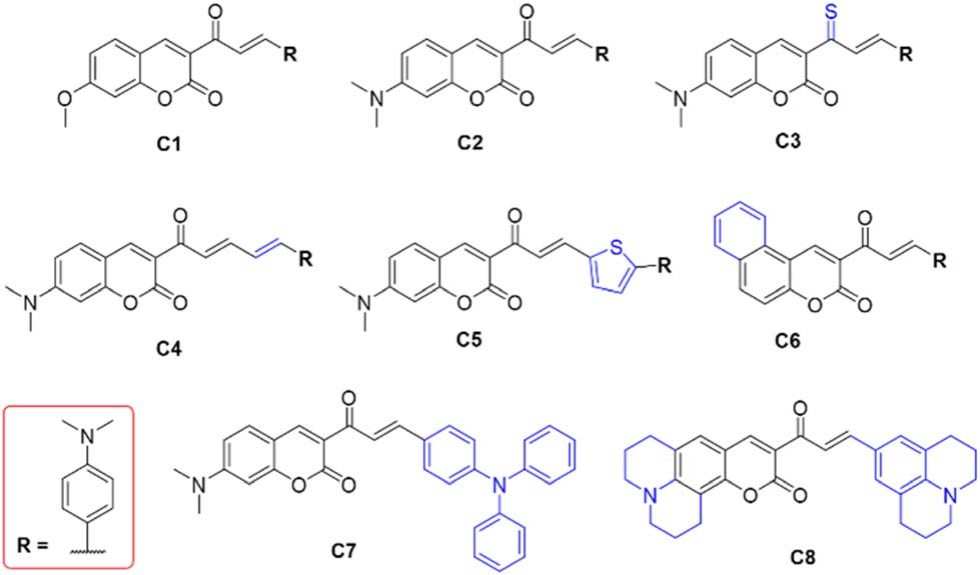
Designed coumarin-based PSs.

Motivated by intriguing photophysical findings of compounds 1–5 in the previous report,^56^ the present research is grounded in a quantum chemical investigation of two coumarin-based scaffolds, denoted as C1 and C2, which correspond to 1–3 and 4–5, respectively (Fig. 1). The molecular design strategy sought to enhance SOC through heavy atom effects by transforming the carbonyl functionality in C2 into a thiocarbonyl moiety (C3) and introducing a thiophene substituent (C5). To extend the one-photon (OP) and two-photon (TP) absorption maxima, the π-conjugation of C2 was expanded, resulting in derivatives C4–C6. In parallel, efforts are directed toward reducing (Δ*E*_S–T_) in C2 by incorporating electron donor and acceptor groups (C5–C8). Importantly, the rationally designed PSs are anticipated to be accessible *via* straightforward synthetic routes employing commercially available reagents. Taken together, this study establishes a rational design of coumarin-based heavy-metal-free PSs for one- and two-photon excited FL imaging as well as PDT applications.

The frontier molecular orbitals and corresponding energy levels of C1–C8 were analyzed at their optimized S_0_ geometries to elucidate the relationship between molecular structure and electronic configuration (Fig. S2). In general, the lowest unoccupied molecular orbital (LUMO) is localized on the coumarin acceptor moiety and partially on the donor unit, whereas the highest occupied molecular orbital (HOMO) is primarily distributed over the donor framework with extension toward the carbonyl groups. The HOMO–LUMO energy gaps (*E*_g_) of C1 and C2 are comparable (3.14–3.24 eV). Structural modification decreases *E*_g_ to 2.87 eV (C3) and 2.88 eV (C5), with intermediate values for C4 (3.01 eV) and C6 (2.98 eV), and only marginal reductions for C7 (3.10 eV) and C8 (3.12 eV). Collectively, these molecular design strategies promote a redshift in OPA, TPA, and emission maxima, thereby enhancing the potential utility of these PSs in FL imaging and PDT.^57^

### One-photon and two-photon absorption

The one-photon absorption (OPA) properties of coumarins C1–C8 were calculated using the TDA TD-DFT method based on the optimized S_0_ geometries, and the results are summarized in Tables 1 and S1. The absorption maxima of C1, C2, and C4–C8 are associated with the S_0_ → S_1_ electronic transition, whereas that of C3 corresponds to the S_0_ → S_2_ transition. In all cases, the electronic configurations are primarily dominated by the HOMO → LUMO excitation, contributing more than 95% to the transition character (see Table S1), and are characterized by the intramolecular charge-transfer (ICT) nature.

**Table 1:** Excited state properties of coumarin-based PSs at S_0_ geometry by TDA TD-DFT method using PBE0/def2-TZVP/CPCM (toluene). OPA wavelength (*λ*_abs_); oscillator strength (*f*); vertical energy (*E*_vt_)

	S_0_ → S_1_			S_0_ → S_2_			S_0_ → S_3_			S_0_ → S_4_		
	*E*_vt_ (eV)	*λ*_abs_ (nm)	*f*	*E*_vt_ (eV)	*λ*_abs_ (nm)	*f*	*E*_vt_ (eV)	*λ*_abs_ (nm)	*f*	*E*_vt_ (eV)	*λ*_abs_ (nm)	*f*
C1	2.64 (2.75)^a^	469 (451)^a^	0.89	3.31	374	0.00	3.49 (∼3.44)^a^	356 (∼360)^a^	0.82	3.75	331	0.11
C2	2.70 (2.67)^a^	459 (464)^a^	1.26	3.17	391	0.40	3.35	370	0.00	3.77 (∼3.78)^a^	328 (∼310)^a^	0.22
C3	1.97	631	0.01	2.39	520	1.23	2.72	455	0.41	3.47	357	0.24
C4	2.53	491	1.66	3.08	402	0.41	3.29	377	0.00	3.50	354	0.28
C5	2.39	518	1.41	3.02	410	0.54	3.30	376	0.00	3.33	372	0.14
C6	2.50	496	0.79	3.20	387	0.46	3.25	382	0.00	3.61	343	0.38
C7	2.63 (2.69)^a^	471 (461)^a^	1.33	3.19	388	0.61	3.34	371	0.00	3.61	344	0.15
C8	2.67	464	1.50	3.39	392	0.47	3.39	366	0.00	3.77	329	0.22

a Experimental absorption wavelength peaks in toluene.^56,57^

The computed absorption maxima for C1, C2 (see Fig. 3a), and C7 show strong agreement with experimental data, exhibiting a mean absolute deviation (MAD) of less than 0.11 eV. The oscillator strength of the S_0_ → S_1_ transition for C1 (*f* = 0.89) is lower than that of C2 (*f* = 1.26), which is consistent with its reduced molar absorption coefficient (∼4.0 × 10^4^ M^−1^ cm^−1^ for C1 compared to ∼7.4 × 10^4^ M^−1^ for C2). This high level of consistency between theory and experiment highlights the suitability of the PBE0 exchange–correlation functional and def2-TZVP basis set for describing the photophysical behavior and excited states of these designed coumarin-based PSs. Relative to C2, the absorption maxima of C3–C7 are red-shifted by approximately 12–61 nm. Additionally, the oscillator strengths of C4–C5 and C7–C8 are enhanced by 0.07–0.40. These improvements are particularly advantageous for applications in FL imaging and PDT (Table 1).

In addition, both C1 and C2 display secondary absorption peaks at shorter wavelengths (∼360 nm and ∼310 nm, respectively) in the experimental spectra, which are well reproduced by the simulations and assigned to the S_0_ → S_3_ and S_0_ → S_4_ transitions, respectively (Tables 1 and S1). Notably, the TD-DFT calculations also predict an additional shoulder absorption band in C2, arising from the S_0_ → S_2_ transition (*λ*_abs_ = 391 nm) with a relatively high oscillator strength (*f* = 0.40), which contributes significantly to the overall absorbance of the absorption peak at 464 nm. A similar spectral feature is observed for C3–C8, wherein the shoulder absorption bands are predominantly attributed to the S_0_ → S_2_ transition (S_0_ → S_3_ for C3), accompanied by another higher-energy absorption at shorter wavelengths assigned to the S_0_ → S_4_ transition.

**Fig. 2:**
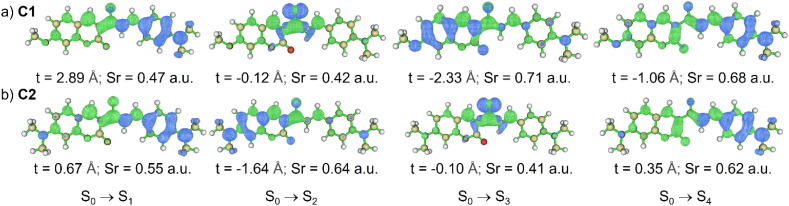
Hole and electron distribution (top) and overlap (bottom) of S_0_ → S_*n*_ transition for (a) C1 and (b) C2 at optimized S_0_ geometry, computed by PBE0/def2-TZVP using CPCM (toluene). Excited state energy is computed by TDA TD-DFT at the same level theory. Blue and green isosurface represent hole and electron distributions, respectively.

To elucidate the nature of the electronic transitions in coumarins C1–C8, hole–electron distribution analyses were carried out, as illustrated in Fig. 2, S3, S5 and S6. For the maxima absorption peaks (S_0_ → S_1_ in C1, C2, and C4–C8; S_0_ → S_2_ in C3), the electron density is primarily localized on the coumarin acceptor moiety, with partial delocalization onto the donor unit, whereas the hole density is mainly distributed over the donor framework and extended toward the carbonyl groups. This spatial separation of electron and hole, together with the positive *t*-index values, is characteristic of intramolecular charge-transfer (^1^ICT) states. In particular, C2, C3, and C8 exhibit positive but small *t*-index values (0.67 Å, 0.25 Å, and 0.26 Å, respectively), suggesting contributions from local excitation (^1^LE) and therefore a hybridized local and charge-transfer (^1^HLCT) character. In contrast, the higher-energy transitions (*e.g.*, S_0_ → S_3_ in C1, and S_0_ → S_2_ in C2 and C4–C8) are dominated by hole and electron densities confined within the coumarin core and carbonyl groups. These states, characterized by negative *t*-index values, can be unambiguously assigned to ^1^LE excitations. Meanwhile, transitions such as S_0_ → S_2_ in C1, S_0_ → S_3_ in C2, S_0_ → S_1_ in C3, and S_0_ → S_3_ in C4–C8 correspond to ^1^n–π* states, consistent with their negligible oscillator strengths and limited contributions to the overall absorption profiles. At higher excitation energies, the S_0_ → S_4_ transitions exhibit distinct differences. In C1, this transition is clearly assigned to a ^1^LE state, consistent with the localized distribution of hole and electron densities in the whole molecule (Fig. 2a). By contrast, C2, C4, C6, and C8 display S_0_ → S_4_ transitions with ^1^HLCT character. Conversely, in C3, C5, and C7, the corresponding states retain a predominantly ^1^ICT nature. This variation highlights the role of substituent effects in tuning n–π*, ICT, HLCT, and LE states within coumarin-based PSs.

**Fig. 3:**
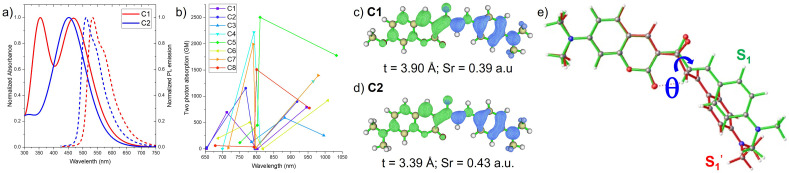
(a) Computed OPA (solid line) and emission (dot line) spectra of C1 and C2 by TDA TD-DFT method using PBE0/def-TZVP/CPCM (toluene); (b) TPA spectra of C1–C8 are computed by B3LYP/def2-TZVP level of theory using PCM (toluene); hole (blue color) and electron (green color) distribution of S_1_ → S_0_ transition of (c) C1 and (d) C2 at S_1_ geometry; (e) overlay of the optimized geometries of ^1^ICT (S_1_, green) and ^1^TICT (
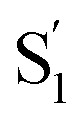
, red) states of C2.

Subsequently, TPA cross sections of the coumarin-based PSs were calculated at the B3LYP/def2-TZVP level of theory in toluene (Fig. 3b and Table 2). The excitation of S_0_ → S_1_ for C1–C8 (except for C3, 778–1779 GM) and S_0_ → S_2_ for C3 (225 GM) characterized by ICT character, is associated with relatively large TPA cross sections in the near-infrared (NIR) region (918–1033 nm) (Table 2). In agreement with experimental measurements, the computed TPA cross section of C1 (∼363 GM) is substantially lower than that of C2 (∼1550 GM). By contrast, the S_0_ → S_2_ transition of C1–C2 and C6–C7, and S_0_ → S_1_ for C3 contributes negligibly to the overall TPA response. In addition, the S_0_ → S_3_ transition of C1–C7 gives rise to pronounced TPA cross sections (451–2219 GM) located in the red and NIR spectral region (719–882 nm). Taken together, these results demonstrate that C1–C8 possess broad and large TPA spanning the red-to-NIR region (Fig. 3b), thereby underscoring their potential utility in two-photon-excited FL imaging and PDT.

**Table 2:** TP absorption properties of PSs are computed by B3LYP/def2-TZVP level of theory using PCM (toluene). Vertical energy (*E*_vt_); TP absorption wavelength (*λ*_TPA_); TP absorbance (*δ*_TPA_)

	S_0_ → S_1_			S_0_ → S_2_			S_0_ → S_3_			S_0_ → S_4_		
	*E*_vt_ (ev)	*λ*_TPA_ (nm)	*δ*_TPA_ (GM)	*E*_vt_ (ev)	*λ*_TPA_ (nm)	*δ*_TPA_ (GM)	*E*_vt_ (ev)	*λ*_TPA_ (nm)	*δ*_TPA_ (GM)	*E*_vt_ (ev)	*λ*_TPA_ (nm)	*δ*_TPA_ (GM)
C1	2.62	946	791	3.09	802	0	3.48	713	696	3.79	654	2
C2	2.70	918	900	3.12	795	0	3.23	768	1155	3.79	654	25
C3	1.78	1393	1	2.49	996	256	2.81	882	592	3.16	785	131
C4	2.57	965	1286	3.06	810	120	3.13	792	2219	3.54	700	0
C5	2.40	1033	1779	3.06	810	2504	3.09	802	451	3.30	751	118
C6	2.46	1008	922	3.03	818	0	3.18	780	506	3.61	687	203
C7	2.53	980	1398	3.11	797	18	3.14	790	1992	3.46	717	17
C8	2.60	954	778	3.10	800	1510	3.13	792	37	3.65	679	62

### Fluorescence emission and dark state

To investigate the fluorescence emission of coumarin-based PSs, the geometries of the first singlet excited states (S_1_) were optimized, and the corresponding excited-state characteristics are summarized in Table 3. C1, C2, (see Fig. 3a), and C7 display emission maxima at 548 nm, 511 nm, and 525 nm, respectively, in close agreement with the experimental observations (MAD < 0.11 eV). Similarly, C4–C6 exhibit emission within the 539–598 nm range. For most of the designed PSs, the S_1_ → S_0_ transition (except for C3) is dominated by ICT character, as illustrated in Fig. 3c, d, S8, S10 and 11a (highly positive *t*-index of hole–electron density distribution). These transitions are associated with relatively high oscillator strengths (*f* = 0.56–1.31), indicative of intense fluorescence emission. In contrast, C3 exhibits a negligible oscillator strength, which is attributed to its ^1^n–π* transition, a characteristic feature of weak-to-non emissive states.

**Table 3:** Excited state properties of coumarin-based PSs at S_1_ geometry by TDA TD-DFT method using PBE0/def2-TZVP/CPCM (toluene). Fluorescence emission wavelength (*λ*_ems_); oscillator strength (*f*); vertical energy (*E*_vt_)

PS	*λ*_ems_ (nm)	*f*	*E*_vt_ (eV)	Transition	*k*_F_ (s^−1^)	*k*_IC_ (s^−1^)
C1	548 (564)^a^	0.63	2.26 (2.20)^a^	L → H (98.4%)	2.9 × 10^8^ (8.2 × 10^7^)^b^	2.9 × 10^9^ (2.8 × 10^9^)^c^
C2	511 (537)^a^	0.90	2.42 (2.31)^a^	L → H (98.0%)	5.3 ×10^8^ (1.6 ×10^8^)^b^	9.0 ×10^8^ (1.6 × 10^9^)^c^
C3	842	0.00	1.47	L → H-1 (98.7%)	6.6 ×10^2^	5.8 ×10^2^
C4	539	1.29	2.30	L → H (97.3%)	4.0 ×10^8^	5.0 ×10^8^
C5	568	1.31	2.18	L → H (97.3%)	4.7 ×10^8^	7.4 × 10^8^
C6	598	0.56	2.07	L → H (98.6%)	2.5 × 10^8^	7.5 ×10^8^
C7	525 (518)^a^	0.89	2.36 (2.39)^a^	L → H (96.4%)	5.0 × 10^8^	7.1 × 10^8^
C8	539	0.88	2.30	L → H (98.3%)	6.9 ×10^7^	1.9 ×10^8^

a Experimental emission wavelength peaks in toluene.^56,57^

b Experimental radiative decay rate (*k*_r_).

c Experimental non-radiative decay rate (*k*_nr_) (see more in details in Table S2).

To gain deeper insight into the excited-state dynamics of the coumarin-based PSs, FL and IC rate constants for the S_1_ → S_0_ transition were computed using Fermi's Golden Rule within the framework of the adiabatic Hessian model.^51^*k*_FL_ and *k*_IC_ are obtained by applying the transition dipole moment (*μ̂*) operator^52^ and non-adiabatic coupling (NAC) operator,^58,59^ respectively as perturbative Hamiltonian operator in eqn (2). For the evaluation of the FL rates, the Tamm–Dancoff approximation (TDA)^60,61^ was employed in conjunction with Voigt line-shape functions,^62^ while the IC rates were determined using the full TD-DFT formalism in combination with nonadiabatic coupling matrix elements (NACMEs) and the electron translation factor (ETF).^63,64^ With the exception of C3, the coumarin-based PSs exhibit relatively high FL rates (6.9 × 10^7^–5.3 × 10^8^ s^−1^), although these remain consistently lower than the corresponding IC rates (1.9 × 10^8^–2.9 × 10^9^ s^−1^). For instance, the FL rate of C1 (2.9 × 10^8^ s^−1^) is lower than that of C2 (5.3 × 10^8^ s^−1^), whereas the IC rate of C1 (2.9 × 10^9^ s^−1^) is notably higher than that of C2 (9.0 × 10^8^ s^−1^). Importantly, both the FL and IC rates of C1 and C2 show strong agreement with the experimentally determined radiative (*k*_r_) and non-radiative (*k*_nr_) decay rates. Specifically, the experimental *k*_r_ of C1 (8.2 × 10^7^ s^−1^) is lower than that of C2 (1.6 × 10^8^ s^−1^), while the experimental *k*_nr_ of C1 (2.8 × 10^9^ s^−1^) exceeds that of C2 (1.6 × 10^9^ s^−1^). In sharp contrast, both the FL and IC rates of C3 are markedly suppressed (5.8–6.6 × 10^2^ s^−1^) relative to the other PSs. This behavior can be attributed to the predominant n–π* character of the S_1_ state in C3, which is well known to give rise to inefficient radiative and non-radiative decay pathways.

In experimental observations,^56^ the fluorescence decay traces of C1 and C2 were well described by a biexponential function, in which the predominant component (>96%) corresponded to a short fluorescence lifetime of 0.31–0.52 ns. Wu *et al.* attributed the minor, longer-lived component (1.8–2.8 ns) to isomers formed *via* photoisomerization; however, this assignment was not unambiguously established.^56^ Our recent studies on BOPAM and coumarin-based fluorophores have demonstrated the presence of a dark charge-transfer (CT) state. Such states may arise through either a ^1^ICT → ^1^CT or ^1^HLCT → ^1^CT transition, both of which can significantly diminish or quench fluorescence emission. Motivated by this, we optimized an alternative excited-state geometry (denoted as 
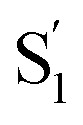
) for C1–C8 (see results in Table 4).

**Table 4:** Excited state properties of coumarin-based PSs at 
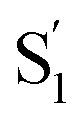
 geometry by TDA TD-DFT method using PBE0/def2-TZVP/CPCM (toluene). Adiabatic energy gap between S_1_ and 
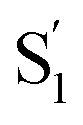
 geometry 
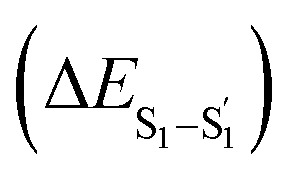
; fluorescence emission (*λ*_ems_); oscillator strength (*f*); vertical energy (*E*_vt_)

PS	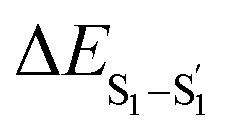	*λ*_ems_ (nm)	*f*	*E*_vt_ (eV)	Transition
C1	0.22	973	0.01	1.27	L → H (97.8%)
C2	0.17	875	0.01	1.42	L → H (97.7%)
C3	0.02	1856	0.00	0.67	L → H (94.5%)
					L → H-1 (3.8%)
C4	0.12	933	0.01	1.33	L → H (97.5%)
C5	0.01	895	0.02	1.38	L → H (97.1%)
					L → H-1 (2.3%)
C6	0.20	1094	0.01	1.13	L → H (97.8%)
C7	−0.01	768	0.02	1.61	L → H (97.1%)
					L → H-1 (2.3%)
C8	0.12	860	0.01	1.44	L → H (97.9%)

Remarkably, the optimized 
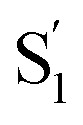
 geometry features a ∼90° torsional twist around the C(=O)–C(=C) bond bridge linking the carbonyl coumarin acceptor to the donor moiety (Fig. 3e). The 
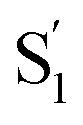
 state was found to emit at longer wavelengths (768 nm), but with negligible oscillator strength (*f* < 0.02). In the 
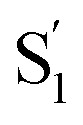
 state, the electron density is localized on the carbonyl coumarin acceptor while the hole is localized on the donor, accompanied by a higher *t*-index and a lower S_r_ index compared to the planar S_1_ state (Fig. S11b). These characteristics are consistent with assignment of 
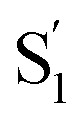
 as a dark twisted ^1^TICT state. For C1 and C2, the adiabatic energy of S_1_ was found to be higher than that of 
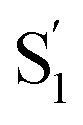
 (Δ*E* = 0.17–0.22 eV), suggesting a possible emissive ^1^ICT → dark ^1^TICT transition. This provides a rational explanation for both the quenched emission and the low experimental FLQYs observed (0.028–0.089). Furthermore, the small Δ*E* values between S_1_ and 
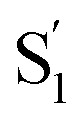
 for C3–C8 (−0.01 to 0.20 eV) indicate that population of the ^1^TICT state is feasible under photoexcitation, further supporting the mechanism of emission quenching.

**Fig. 4:**
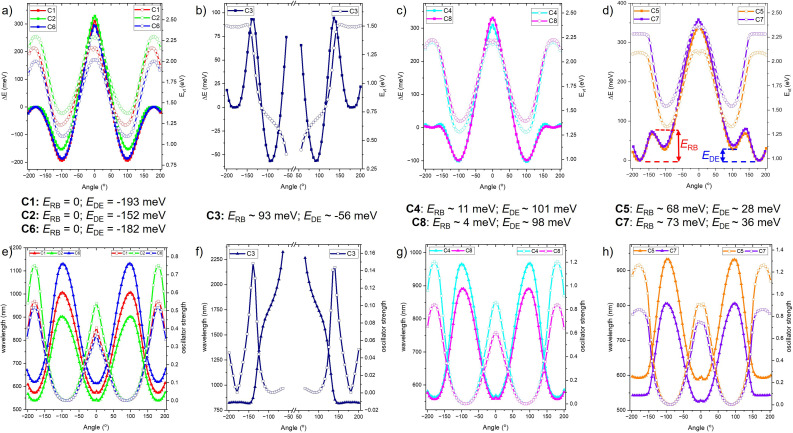
The change of minimal energy (Δ*E*) and vertical energy (*E*_vt_) of 
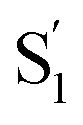
 geometry under angle (*θ*) scan of (a) C1, C2 and C6; (b) C3; (c) C4 and C8; (d) C5 and C7 by TD-DFT method using PBE0/def2-TZVP/CPCM (toluene). The change of wavelength and oscillator strength of 
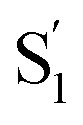
 geometry under angle (*θ*) scan of (e) C1, C2 and C6; (f) C3; (g) C4 and C8; (h) C5 and C7. Rotation barrier (*E*_RB_) and driving energy (*E*_DE_).

Theoretically, the emissive ^1^ICT → dark ^1^TICT transition in C1–C8 can be elucidated by constructing the S_1_ potential energy surface (PES) as a function of donor group rotation (*θ*). Two parameters are particularly critical for evaluating the degree of TICT formation:^24^ the rotation barrier (*E*_RB_) and the driving energy (*E*_DE_) (Fig. 4a–d). For C1, C2, and C6, the PES reveals a vanishing *E*_RB_ and a significantly negative *E*_DE_ (−152 to −193 meV) (Fig. 4a). This combination strongly favors the ^1^ICT → ^1^TICT transition, leading to substantial population of the dark ^1^TICT state and, consequently, pronounced fluorescence quenching. By contrast, C3 displays a finite positive *E*_RB_ (∼93 meV) alongside a moderately negative *E*_DE_ (∼−56 meV). Nonetheless, nonradiative deactivation of C3 from the S_1_ state is primarily governed by efficient ISC, as discussed in the subsequent section. C4 and C8 present comparatively small barriers to rotation (*E*_RB_ = 4–11 meV) together with strongly negative *E*_DE_ values (−98 to −101 meV). These features suggest partial population of the ^1^TICT state and, therefore, incomplete fluorescence quenching. In contrast, the PES profiles of C5 and C7 show both positive *E*_RB_ (68 to 73 meV) and positive *E*_DE_ (28 to 36 meV). This energetic landscape indicates that the TICT state is energetically inaccessible, implying that fluorescence quenching *via* the ^1^ICT → ^1^TICT channel does not occur for these systems.

In addition, analysis of the vertical excitation energy (*E*_vt_), oscillator strength, and emission wavelength as a function of *θ* reveals strong angular dependence (Fig. 4). Specifically, *E*_vt_ and oscillator strength attain their maxima and minima near ±180° and ±90°, respectively, whereas the emission wavelength exhibits the opposite trend, with longest values around ±90° and shortest values near ±180°. Excluding C3, the substitution pattern of donor groups (*e.g.*, C4–C5 and C7–C8 compared to C1–C2 and C6) effectively weakens the electron-donating capacity. This attenuation of donor strength reduces the propensity for the ^1^ICT → ^1^TICT transition, thereby suppressing TICT population and mitigating fluorescence quenching.

### Type II photoreaction mechanism

In the experimental analysis, C2 demonstrates a high triplet state QY as well as singlet oxygen QY. To further elucidate the triplet excited states and ISC processes of C1–C8, the geometries of the T_1_, T_2_, and T_3_ states were optimized using the TDA TD-DFT method. The adiabatic energy of the S_1_ state was found to be higher than that of T_1_ and T_2_, but lower than that of T_3_ (Table 5 and S5), suggesting possible ISC pathways *via* the S_1_ → T_1_ and S_1_ → T_2_ channels. Importantly, the calculated vertical excitation energies of the T_1_ and T_2_ states for C1 and C2 showed strong correspondence with experimental data, consistent with the observation of two transient absorption peaks at 530 nm and 690 nm for C1, and at 520 nm and 770 nm for C2 (MAD = 0.08–0.13 eV for C1; 0.13–0.24 eV for C2). Except for C3, the hole–electron density distribution of the T_1_ state is primarily localized on the donor groups with extension into the carbonyl moiety, indicative of a ^3^LE character. In contrast, the T_2_ state exhibits hole–electron density localized within the carbonyl coumarin fragment, denoting a distinct ^3^LE′ character. For C3, the T_1_ and T_2_ states are instead assigned to ^3^π–π* and ^3^n–π* transitions, respectively. Overall, the S_1_ → T_1_/T_2_ transitions of C1–C8 involve changes in orbital type, thereby facilitating efficient ISC processes according to El-Sayed's rule.

**Table 5:** Adiabatic energy gap (Δ*E*_S–T_) and ISC constant rate (*k*_ISC_) between first singlet (S_1_) and triplet (T_*n*_) excited states by TDA TD-DFT method using PBE0/def2-TZVP/CPCM (toluene) and Fermi's Golden Rule expression (see computational detail and discussion). Computed FL QY (*Φ*_F_) and triplet QY (*Φ*_T_) (experimental results)

	S_1_ → T_1_		S_1_ → T_2_		Total		
	Δ*E*_S–T_ (eV)	*k*_ISC_ (s^−1^)	Δ*E*_S–T_ (eV)	*k*_ISC_ (s^−1^)	*k*_ISC_ (s^−1^)	*Φ*_F_	*Φ*_T_
C1	0.62	1.13 × 10^8^	0.10	2.00 × 10^7^	1.33 × 10^8^	0.087 (0.028)	0.043 (0.25)
C2	0.67	1.24 × 10^8^	0.33	2.90 × 10^7^	1.53 × 10^8^	0.335 (0.089)	0.098 (0.52)
C3	0.36	7.42 × 10^10^	0.23	1.94 × 10^12^	2.01 × 10^12^	0.000	1.000
C4	0.86	5.26 × 10^6^	0.25	1.58 × 10^6^	6.84 × 10^6^	0.441	0.007
C5	0.76	1.48 × 10^6^	0.12	2.40 × 10^4^	1.50 × 10^6^	0.388	0.001
C6	0.51	5.16 × 10^7^	0.15	4.60 × 10^5^	5.20 × 10^7^	0.238	0.049
C7	0.60	3.62 × 10^4^	0.26	6.40 × 10^3^	4.26 × 10^4^	0.413 (0.201)	0.000
C8	0.64	1.02 × 10^6^	0.33	1.34 × 10^6^	2.36 × 10^6^	0.264	0.009

Within the framework of Fermi's Golden Rule, the perturbation governing the ISC rate constant (*k*_ISC_) arises from the spin–orbit coupling (SOC) operator. The purely electronic SOC operator is described by the Breit–Pauli spin–orbit Hamiltonian,^65^ which under the Franck–Condon approximation is independent of nuclear coordinates:

3 *Ĥ*^(0)^_SOC_ = *ζ*(*r*)*L̂*×*Ŝ*

where *ζ*(*r*) denotes the SOC parameter, which scales approximately with *Z*^4^, *L̂* and *Ŝ* represent the orbital and spin angular momentum operators, respectively. However, in heavy-atom-free PSs, Herzberg–Teller (HT) vibronic effects^66^ play a critical role in modulating the SOC operator and thereby influencing ISC efficiency.^65,67^ To account for such vibronic contributions, the SOC operator is expanded to include the first-order term in a Taylor expansion with respect to the normal-mode nuclear displacement coordinate (*Q*_*k*_):^65^4
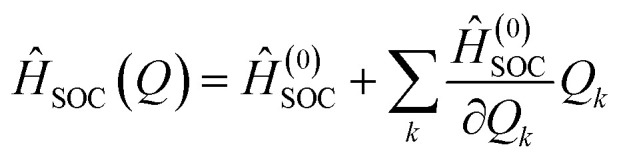


An additional aspect of the theory involves the relationship between the initial (*Q*_i_) and final (*Q*_f_) vibrational coordinates, which are connected through the Duschinsky relation:

5 *Q*_i_ = *J*·*Q*_f_ + *K*

where *J* is the Duschinsky rotation matrix describing mode mixing between initial and final states, and *K* is the displacement vector accounting for equilibrium geometry shifts upon electronic excitation.

Subsequently, the *k*_ISC_ for the S_1_ → T_1_/T_2_ transitions of C1–C8 were computed using Fermi's Golden Rule within the framework of the adiabatic Hessian model, incorporating both Franck–Condon (FC) and Herzberg–Teller (HT) contributions.^66^ The RIJCOSX and RI-SOMF(1X) approximation^48,68,69^ were employed in conjunction with the consideration of Duschinsky rotation effects^70,71^ for the ISC rate calculations. Scalar relativistic effects were accounted for using the zero-order relativistic approximation (ZORA).^72^ Furthermore, the ZORA-def2-TZVP basis sets were utilized within the TDA framework to ensure accurate treatment of electronic excitations.

Except for C3 and C8, the ISC rate for the S_1_ → T_1_ channel is significantly higher than that for the S_1_ → T_2_ channel (Table 4), indicating that the ^1^ICT → ^3^LE transition constitutes the primary ISC pathway. In the case of C8, the ISC rates of the S_1_ → T_1_ and S_1_ → T_2_ channels are comparable, suggesting a competitive population of both ^1^ICT → ^3^LE and ^1^ICT → ^3^LE′ transitions. Each ISC rate includes contributions from three triplet sublevels (*M*_s_ = 0, +1, −1). The rates associated with *M*_s_ = ±1 are equivalent and consistently exceed those for *M*_s_ = 0, which demonstrates their principal contribution to the overall ISC rate. Importantly, the HT effect dominates the ISC process for these sublevels, accounting for more than 98% of the ISC rate. This highlights the critical role of the HT effect in facilitating ISC, particularly in heavy-atom-free PSs, in contrast to the FL rates (see Table S4 and Fig. S9). C3 exhibits a markedly higher ISC rate compared to other coumarin-based PSs, both in total and across sublevels for the S_1_ → T_1_ and S_1_ → T_2_ channels. This enhancement arises from the heavy-atom effect of the sulfur atom. For the dominant sublevels (*M*_s_ = ±1), the contribution of the HT effect is minimal (4.4–7.7%), thereby confirming its negligible role in heavy-atom-based PSs.

To evaluate the FL emission and ISC processes of C1–C8, the theoretical FL QY (*Φ*_F_) and triplet QY (*Φ*_T_) were calculated using eqn (6) and (7), with the results summarized in Table 4:6
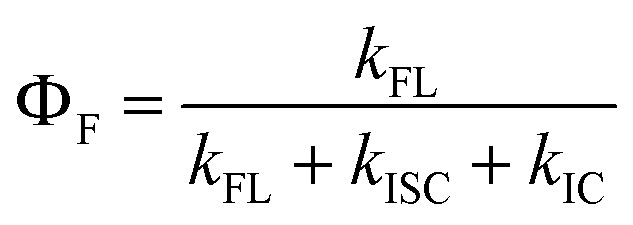
7
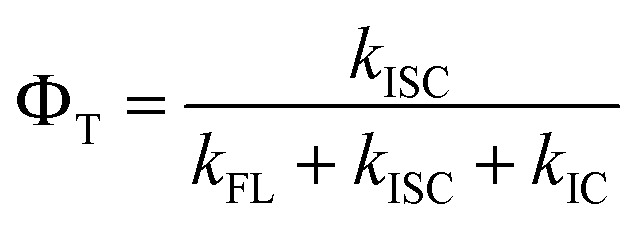


**Fig. 5:**
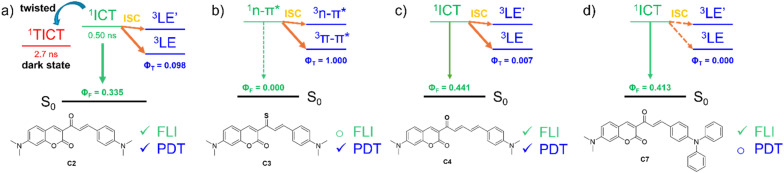
Illustration for (non-)radiative decay process of (a) C2, (b) C3, (c) C4 and (d) C7 along with potential application for FL imaging (FLI) and PDT.

For C1, C2, and C6, the theoretical values of *Φ*_F_ and *Φ*_T_ may be reduced in the presence of non-radiative decay through the ^1^ICT → ^1^TICT transition. In the absence of this pathway, the calculated *Φ*_F_ of C1 (0.087) is approximately 3.5-fold lower than that of C2 (0.335), consistent with the experimental trend (0.028 for C1 and 0.089 for C2). Similarly, the calculated *Φ*_T_ of C1 (0.043) is nearly twofold lower than that of C2 (0.089), again reproducing the experimental trend (0.25 for C1 and 0.52 for C2). For C6, the theoretical *Φ*_F_ and *Φ*_T_ are 0.238 and 0.049, respectively, highlighting its potential for dual applications in both FL imaging and PDT (Fig. 5a). In the case of C3, the ISC process overwhelmingly dominates, resulting in *Φ*_T_ = 1.0 and *Φ*_F_ = 0.0. This feature makes C3 particularly suitable for highly efficient PDT through both Type I and Type II mechanisms (Fig. 5b). By contrast, C4 and C8 exhibit slightly higher *Φ*_F_ values (0.264–0.440) with lower *Φ*_T_, suggesting their preferential application in FL imaging (Fig. 5c). It should also be noted that partial population of the ^1^ICT → ^1^TICT transition may contribute to these systems. Finally, for C5 and C7, the exceptionally high fluorescence yields (0.388–0.441) combined with negligible triplet yields (0.000–0.001), and the absence of ^1^TICT state population, identify these compounds as optimal candidates for fluorescence imaging applications exclusively (Fig. 5d). Overall, these findings demonstrate how subtle variations in substituent effects and electronic configurations across C1–C8 dictate the balance between FL and ISC. Such tunability is particularly valuable for tailoring coumarin-based systems toward specific applications: heavy-atom incorporation drives high triplet yields for PDT, while suppression of non-radiative pathways (*e.g.*, ^1^ICT → ^1^TICT) favors fluorescence for imaging.

## Conclusions

This study establishes a detailed quantum-chemical framework for understanding and controlling the photophysics of coumarin-based PSs. Through Fermi's Golden Rule, fluorescence (*k*_FL_), internal conversion (*k*_IC_), and intersystem crossing (*k*_ISC_) rates were quantitatively determined, allowing direct prediction of quantum yields in agreement with experiment. The comparative analysis highlights three functional regimes: (i) heavy-atom substitution (C3) maximizes ISC, producing an ideal PDT sensitizer; (ii) donor–acceptor tuning (C1, C2, and C6) balances radiative and ISC pathways, offering dual-functionality; and (iii) suppression of ^1^TICT state formation (C5 and C7) ensures strong fluorescence with negligible triplet generation, optimal for imaging. Furthermore, Herzberg–Teller vibronic coupling is identified as the principal driver of ISC in heavy-atom-free systems, while its role is negligible in heavy-atom-based analogues.

In addition to favorable fluorescence and ISC properties, the coumarin derivatives display broad and enhanced two-photon absorption (TPA) responses in the red-to-NIR region. This feature directly addresses the penetration-depth limitations of OPA and improves the prospects for deep-tissue imaging and PDT under clinically relevant excitation conditions. Taken together, these findings define clear structure–property–function correlations and establish rational design rules for next-generation heavy-metal-free PSs, bridging theoretical modeling with biomedical application.

## Conflicts of interest

There are no conflicts to declare.

## Supplementary Material

RA-016-D5RA07339A-s001

## Data Availability

Additional data are available on request. The authors declare that the data supporting the findings of this study are available within the paper and its supplementary information (SI). Supplementary information: excited state properties, hole–electron distribution, and rate constants. See DOI: https://doi.org/10.1039/d5ra07339a.
