# Supplementary material for: Quantum chemical investigation and molecular design of coumarin-based heavy-metal-free photosensitizers for one- and two-photon excited fluorescence imaging and photodynamic therapy
Source: RSC Adv. 2026 Jan 19;16(5):4079–89. doi: 10.1039/d5ra07339a (PMC12814400; doi:10.1039/d5ra07339a)
Supplement: RA-016-D5RA07339A-s001 [file RA-016-D5RA07339A-s001.pdf]

Supplementary Information (SI) for RSC Advances.
This journal is © The Royal Society of Chemistry 2026

Supporting information of

Quantum Chemical Investigation and Molecular Design of Coumarin-based Heavy-metal-free Photosensitizers for One- and Two-Photon Excited Fluorescence Imaging and Photodynamic Therapy

Thanh Chung Pham*[a], Dung Ngoc Tran,[b] Van Trang Nguyen,[a] Van Thong Pham,[c] Dai Lam Tran[b] Songyi Lee[d,e]

[a]Institute of Materials Science, Vietnam Academy of Science and Technology, 18 Hoang Quoc Viet, Cau Giay, Hanoi, Vietnam.

[b]Faculty of Chemistry, Hanoi National University of Education, Hanoi, Vietnam

[c]R&D Center, Vietnam Education and Technology Transfer JSC, Cau Giay, Hanoi, Vietnam

[d] Industry 4.0 Convergence Bionics Engineering, Pukyong National University, Busan 48513, Korea.

[e] Department of Chemistry, Pukyong National University, Busan 48513, Korea

Corresponding author: ptchung@ims.vast.vn

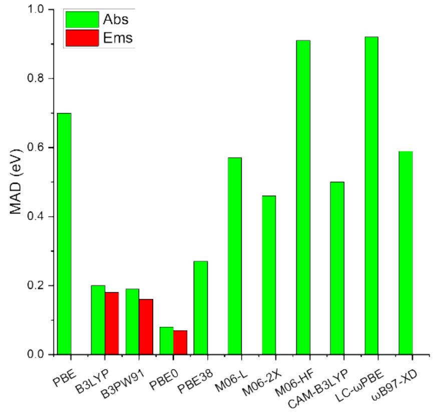

Figure S1. Comparison absorption TDA TD-DFT absorption/emission energies for **C1**, **C2** and **C7** against experiment using representative functionals.

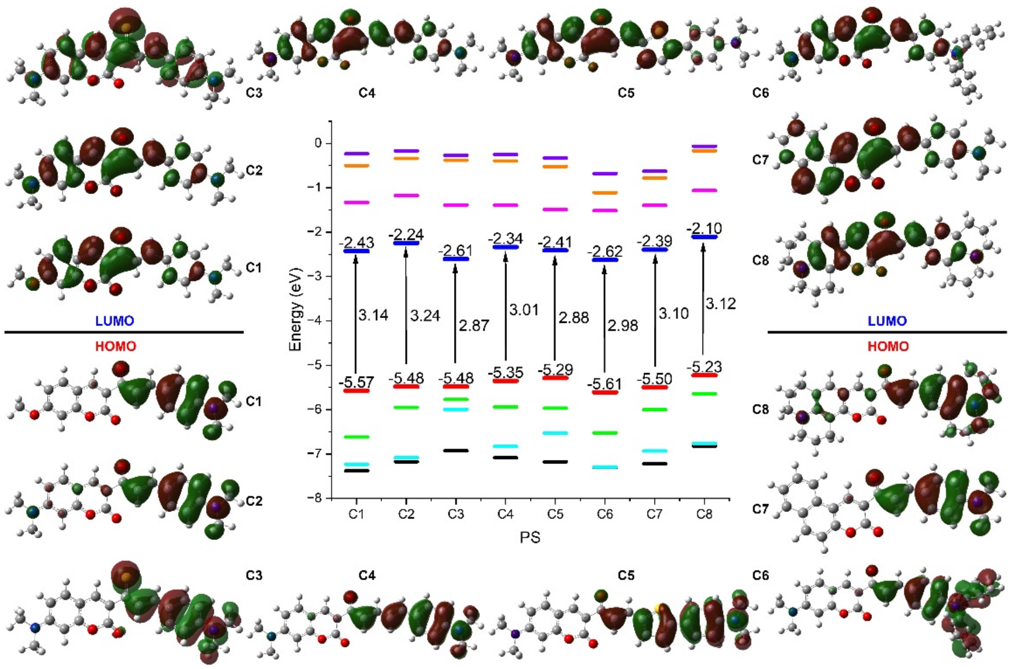

Figure S2. HOMO and LUMO image (around) along with diagram of MO energy levels of **C1-C8** at S_0 geometry.

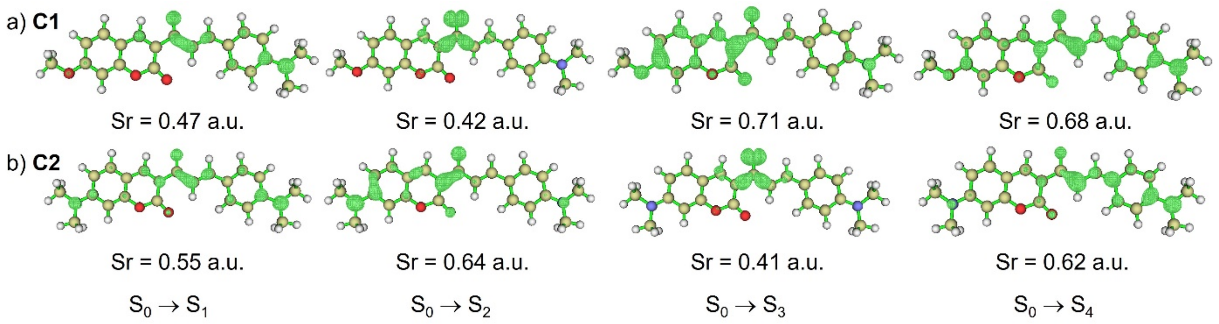

Figure S3. Hole and electron overlap of $S_0 \rightarrow S_n$ transition for (a) **C1** and (b) **C2** at optimized S_0 geometry.

Table S1. Computed OPA properties of **C1-C8**. Fluorescence emission wavelength (λ_{abs}); oscillator strength (*f*); vertical energy (E_{vt}); [a]Experimental OPA wavelength peaks in toluene.[1, 2]

**PSs**	λ_{abs} (nm)	*f*	E_{vt} (ev)	transition state		
**C1**	469 (451)[a]	0.8879	2.64 (2.75)	H → L (98.6 %)		$S_0 \rightarrow S_1$
	374	0.0000	3.31	H-2 → L (94.7 %)	H-2 → L+1 (2.5 %)<br>H-2 → L+5 (2.1 %)	$S_0 \rightarrow S_2$
	356 (~360)[a]	0.8189	3.49 (~3.44)	H-1 → L (76.2 %)	H → L+1 (21.2 %)	$S_0 \rightarrow S_3$
	331	0.1087	3.75	H → L+1 (76.7 %)	H-1 → L (20.3 %)	$S_0 \rightarrow S_4$
**C2**	459 (464)[a]	1.2588	2.70 (2.67)	H → L (95.9 %)	H-1 → L (3.0 %)	$S_0 \rightarrow S_1$
	391	0.4033	3.17	H-1 → L (92.7 %)	H → L (3.4 %)<br>H → L +1 (2.3 %)	$S_0 \rightarrow S_2$
	370	0.0000	3.35	H-2 → L (94.6 %)	H-2 → L+1 (2.3 %)<br>H-2 → L+5 (2.2 %)	$S_0 \rightarrow S_3$
	328 (~310)[a]	0.2245	3.77 (~3.78)	H → L+1 (95.3 %)	H-1 → L (2.4 %)	$S_0 \rightarrow S_4$
**C3**	631	0.0092	1.97	H-1 → L (49.9 %)	H-2 → L (47.6 %)	$S_0 \rightarrow S_1$
	520	1.2318	2.39	H → L (93.6 %)	H-1 → L (4.4 %)	$S_0 \rightarrow S_2$
	455	0.4088	2.72	H-2 → L (48.0 %)	H-1 → L (44.1 %)<br>H → L (5.6 %)	$S_0 \rightarrow S_3$
	357	0.2400	3.47	H → L +1 (84.8 %)	H-1 → L+1 (11.9 %)	$S_0 \rightarrow S_4$
**C4**	491	1.6554	2.53	H → L (96.1 %)	H-1 → L (2.8 %)	$S_0 \rightarrow S_1$
	402	0.4059	3.08	H-1 → L (91.2 %)	H → L+1 (3.6 %)<br>H → L (3.1 %)	$S_0 \rightarrow S_2$
	377	0.0034	3.29	H-3 → L (94.9 %)		$S_0 \rightarrow S_3$
	354	0.2816	3.50	H → L+1 (94.6 %)	H-1 → L (3.9 %)	$S_0 \rightarrow S_4$
**C5**	518	1.4121	2.39	H → L (96.3 %)	H-1 → L (2.1 %)	$S_0 \rightarrow S_1$
	410	0.5424	3.02	H-1 → L (92.0 %)	H → L+1 (3.3 %)<br>H → L (2.6 %)	$S_0 \rightarrow S_2$
	376	0.0020	3.30	H-3 → L (94.8 %)		$S_0 \rightarrow S_3$
	372	0.1366	3.33	H → L+1 (94.1 %)	H-1 → L (3.8 %)	$S_0 \rightarrow S_4$
**C6**	496	0.7926	2.50	H → L (98.4 %)		$S_0 \rightarrow S_1$
	387	0.4590	3.20	H-1 → L (92.2 %)	H → L+1 (5.1 %)	$S_0 \rightarrow S_2$
	382	0.0000	3.25	H-3 → L (94.8 %)	H-3 → L+1 (4.7 %)	$S_0 \rightarrow S_3$
	343	0.3826	3.61	H → L+1 (89.6 %)	H-1 → L (5.4%)<br>H → L+2 (2.5 %)	$S_0 \rightarrow S_4$
**C7**	471 (461)[a]	1.3399	2.63 (2.69)	H→ L (94.6 %)	H-1→ L (3.1 %)	$S_0 \rightarrow S_1$
	388	0.6083	3.19	H-1→ L (88.6 %)	H→ L+1 (4.5 %)<br>H→ L (3.5 %)	$S_0 \rightarrow S_2$
	371	0.0003	3.34	H-3→ L (94.9 %)		$S_0 \rightarrow S_3$
	344	0.1480	3.61	H → L+1 (88.8 %)	H-1 → L (4.0 %)	$S_0 \rightarrow S_4$
**C8**	464	1.5000	2.67	H → L (95.4 %)	H-1 → L (2.9 %)	$S_0 \rightarrow S_1$
	392	0.4725	3.17	H-1 → L (86.1 %)	H → L+1 (8.1 %)<br>H→ L (2.7 %)	$S_0 \rightarrow S_2$
	366	0.0004	3.39	H-4 → L (94.4 %)	H-4 → L+1 (6.6 %)	$S_0 \rightarrow S_3$
	329	0.2156	3.77	H → L+1 (86.8 %)	H-1 → L (94.4 %)	$S_0 \rightarrow S_4$

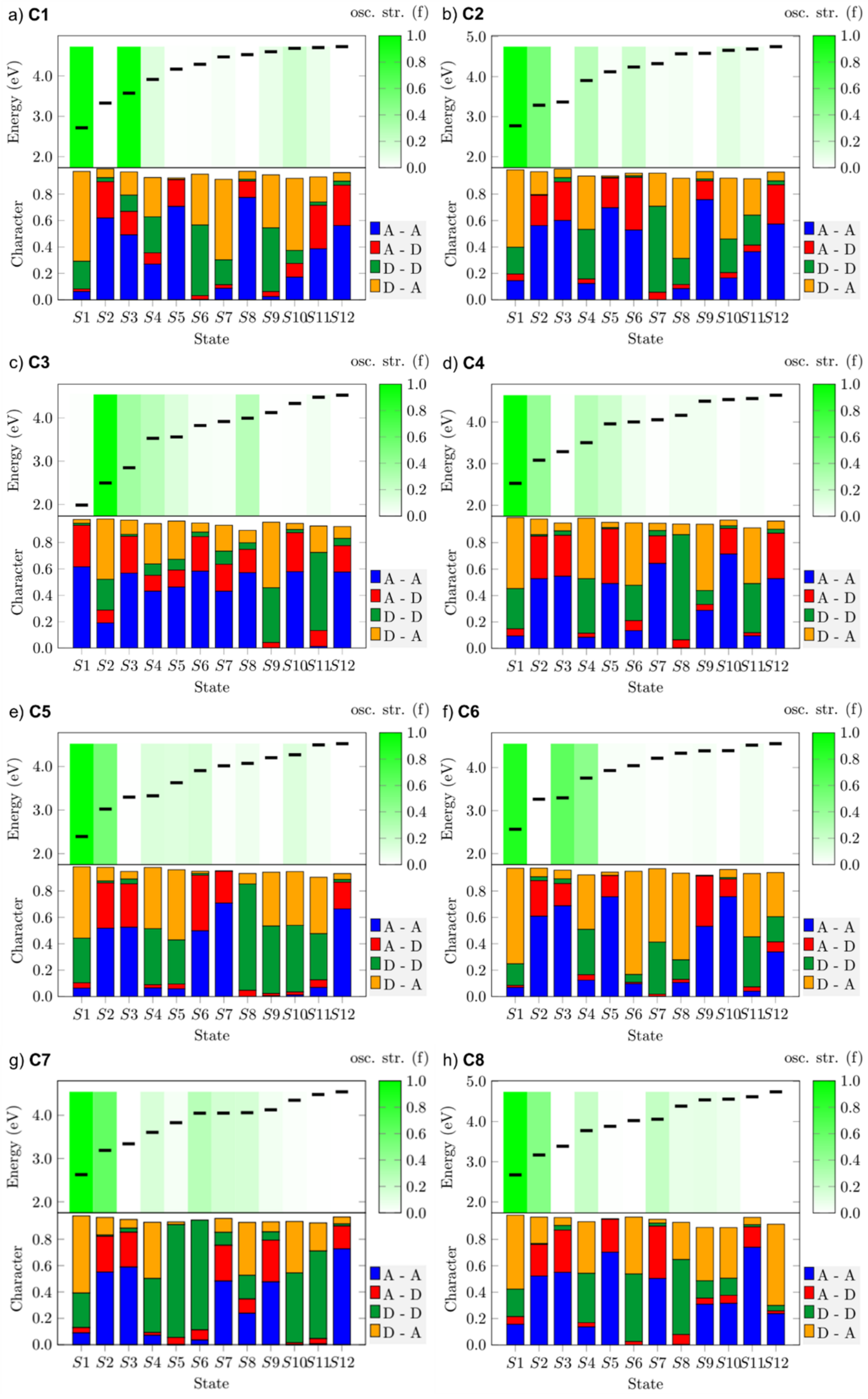

Figure S4. (a-h) Donor (D) and acceptor (A) fragment-based analysis of excited states ($S_1 – S_{12}$) of **C1-C8**. Charge transfer from D/A to D/A (D/A – D/A).

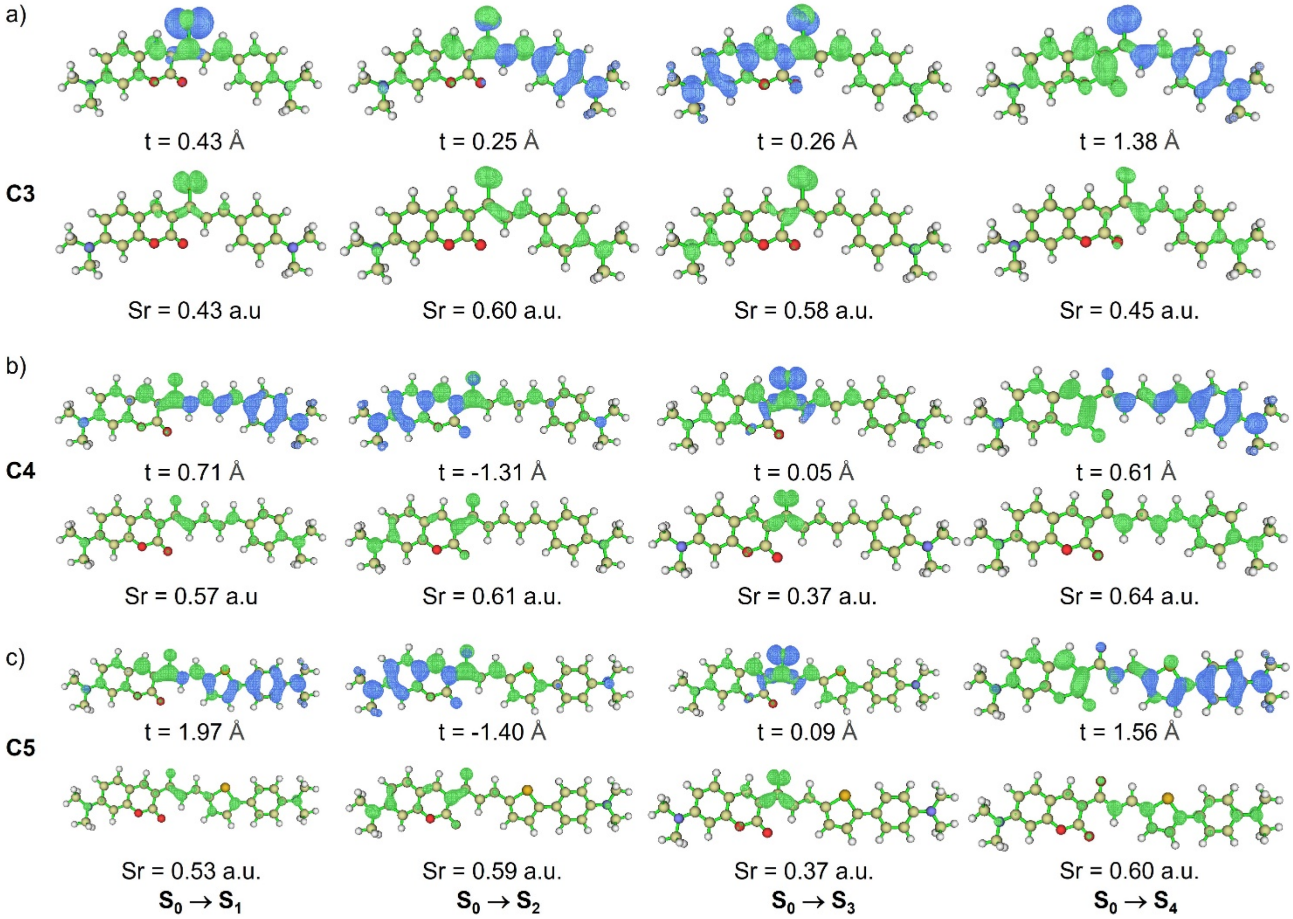

Figure S5. Hole and electron distribution (top) and overlap (bottom) of $S_0 \rightarrow S_n$ transition for (a) **C3**, (b) **C4** and (c) **C5** at optimized S_0 geometry. Blue and green isosurface represent hole and electron distributions, respectively.

Table S2. TPA absorption properties of PSs are computed by **CAM-B3LYP**/def2-TZVP.

	$S_0 \rightarrow S_1$			$S_0 \rightarrow S_2$			$S_0 \rightarrow S_3$			$S_0 \rightarrow S_4$		
	E_{vt} (ev)	λ_{TPA} (nm)	δ_{TPA} (GM)	E_{vt} (ev)	λ_{TPA} (nm)	δ_{TPA} (GM)	E_{vt} (ev)	λ_{TPA} (nm)	δ_{TPA} (GM)	E_{vt} (ev)	λ_{TPA} (nm)	δ_{TPA} (GM)
**C1**	3.34	742	183	3.52	704	0	3.96	626	201	4.42	561	157
**C2**	3.34	742	286	3.54	700	0	3.75	661	381	4.46	556	184
**C3**	2.03	1221	0	2.90	855	117	3.23	768	122	4.17	595	413
**C4**	3.17	782	193	3.50	708	4	3.70	670	620	4.28	579	561
**C5**	3.04	816	231	3.51	706	3	3.65	679	581	4.14	599	869
**C6**	3.24	765	183	3.49	710	0	3.65	679	194	4.31	575	205
**C7**	3.25	763	206	3.53	702	0	3.69	672	701	4.15	598	40
**C8**	3.22	770	92	3.56	696	2	3.63	683	726	4.30	577	258

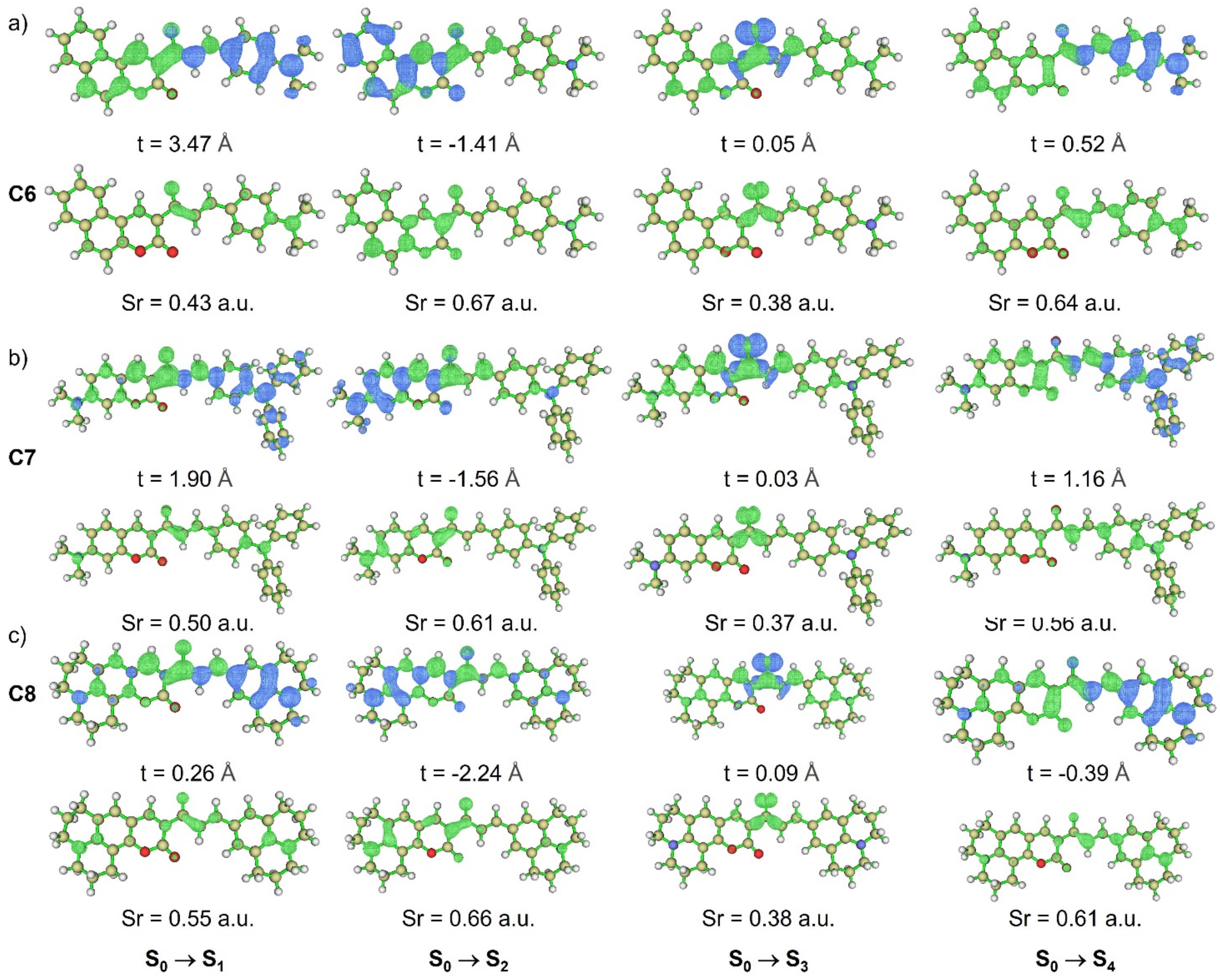

Figure S6. Hole and electron distribution (top) and overlap (bottom) of $S_0 \rightarrow S_n$ transition for (a) **C6**, (b) **C7** and (c) **C8** at optimized S_0 geometry. Blue and green isosurface represent hole and electron distributions, respectively.

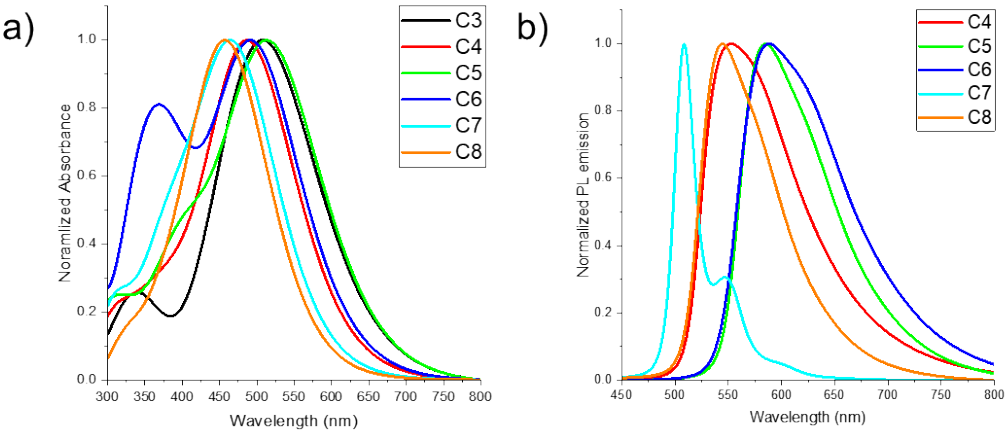

Figure S7. a) Computed absorption spectra and b) computed emission spectra of **C3 – C8** by TDA TD-DFT method using PBE0/def2-tzvp level theory in PCM (toluene).

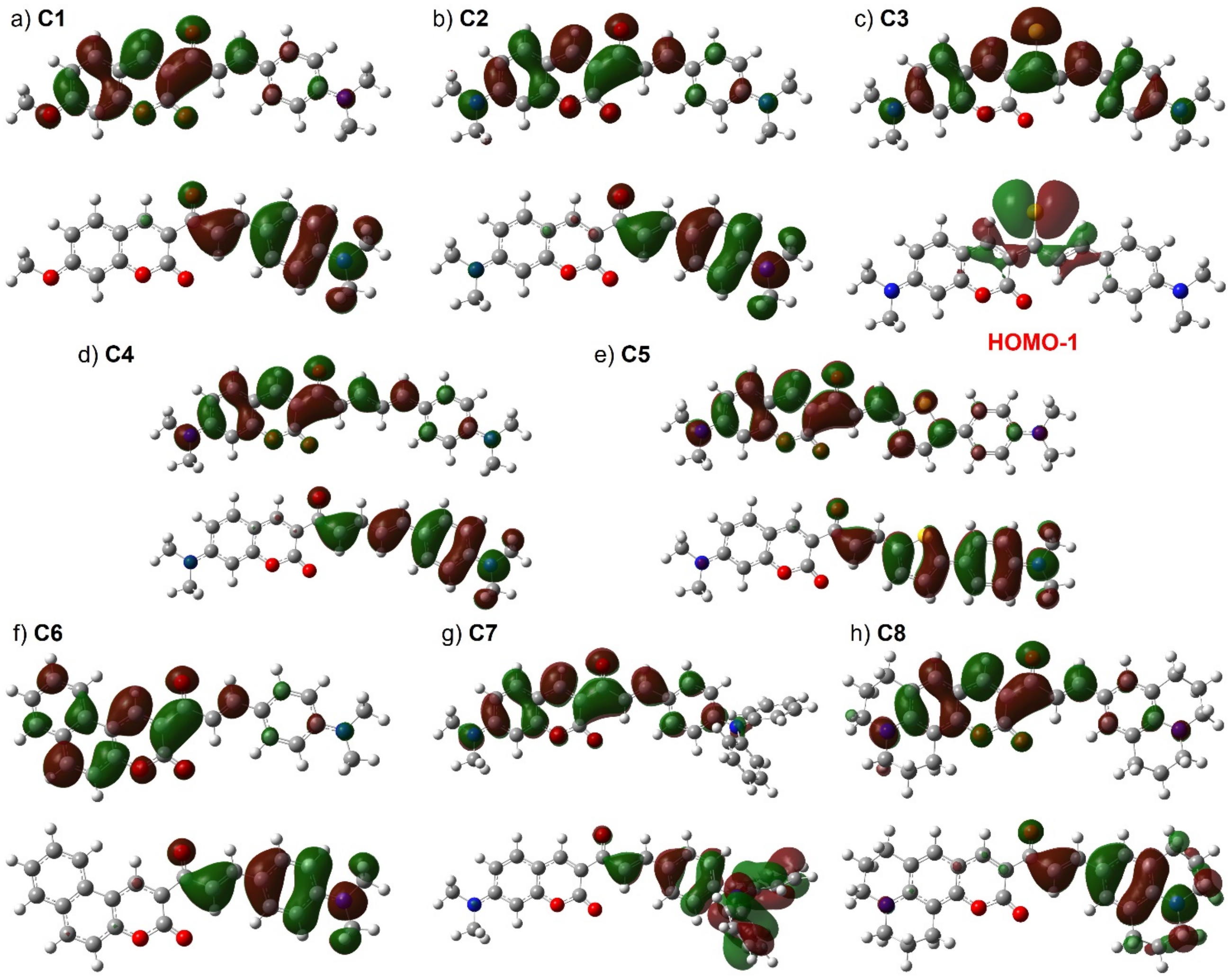

Figure S8. The HOMO (bottom) and LUMO (top) image of (a-h) **C1-C8** at optimized planar S_1 geometry (HOMO-1 for **C3**).

Table S3. Photophysical properties of coumarin **1 – 5** in toluene in the previous report.[1] Fluorescence emission quantum yield (Φ_{FL}); florescence lifetime (τ); radiative decay rate (k_r); non-radiative decay rate (k_{nr}).

Compd	Φ_{FL}	Φ_{FL}	τ_1 (ns)	τ_2 (ns)	τ (ns)	τ (ns)	k_r (s^{-1})	k_{nr} (s^{-1})
**1**	0.031		0.35 (99.34%)	2.3 (0.66%)	0.36			
**2**	0.028	0.028	0.31(99.25%)	1.8 (0.75%)	0.32	0.35	8.19×10^7	2.81×10^9
**3**	0.026		0.34 (99.30%)	2.3 (0.70%)	0.35			
**4**	0.084	0.089	0.48 (96.26%)	2.8 (3.74%)	0.57	0.57	1.55×10^8	1.60×10^9
**5**	0.093		0.52 (97.43%)	2.6 (2.57%)	0.57			

Table S4. Fluorescence rate constants (k_F) of **C1-C8** and contribution of Herzberg-Teller.

State	**C1**	**C2**	**C3**	**C4**	**C5**	**C6**	**C7**	**C8**
k_F (s^{-1})	4.3×10^8	6.5×10^8	2.4×10^5	6.1×10^8	5.3×10^8	2.5×10^8	6.9×10^8	5.2×10^8
HT (%)	12.4	7.0	80.5	-1.1	3.4	-3.3	2.6	-8.2

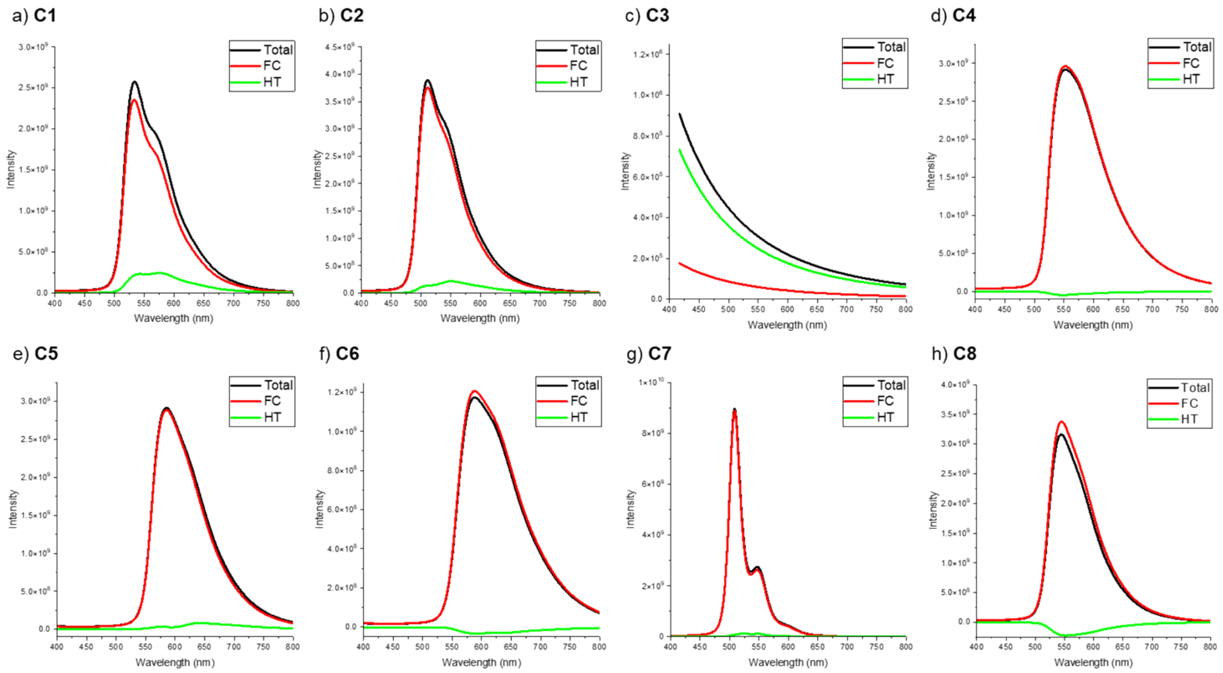

Figure S9. Fluorescence emission spectra of (a-h) **C1-C8** with contribution of Franck-Condon and Herzberg-Teller.

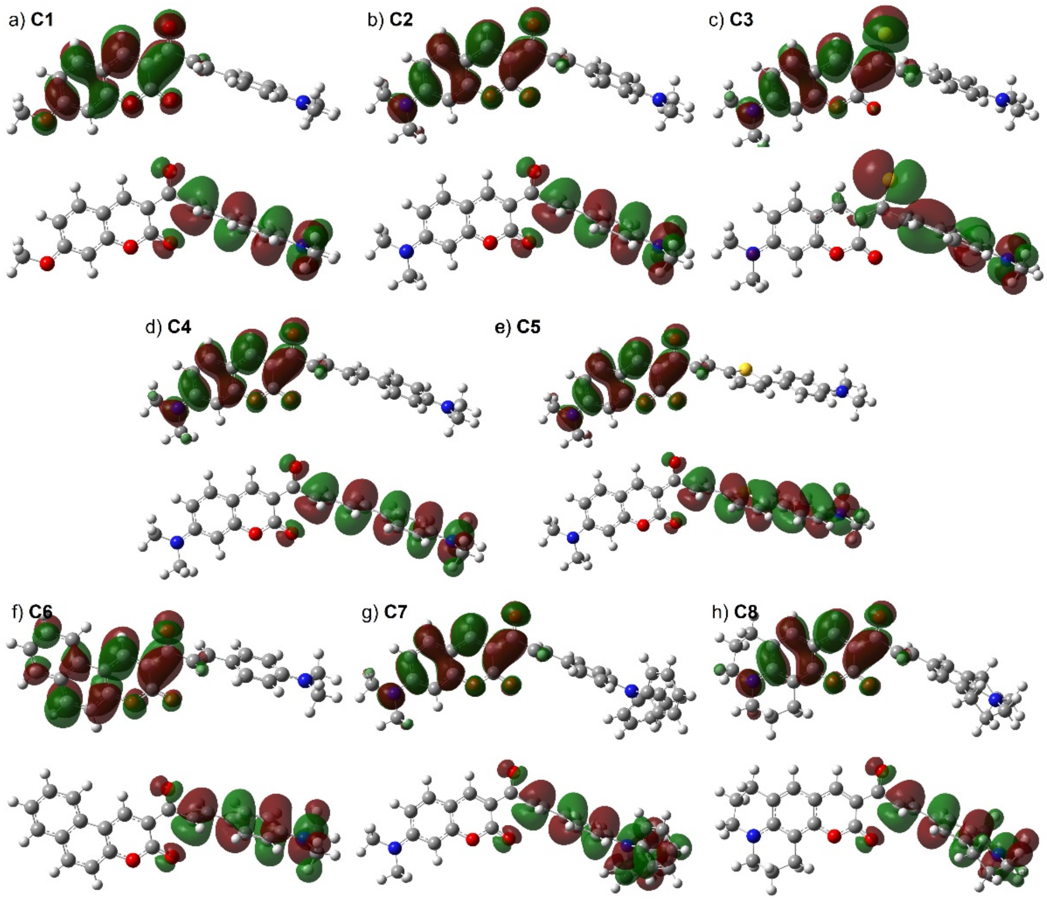

Figure S10. The HOMO (bottom) and LUMO (top) image of (a-h) **C1-C8** at optimized twisted S_1’ geometry.

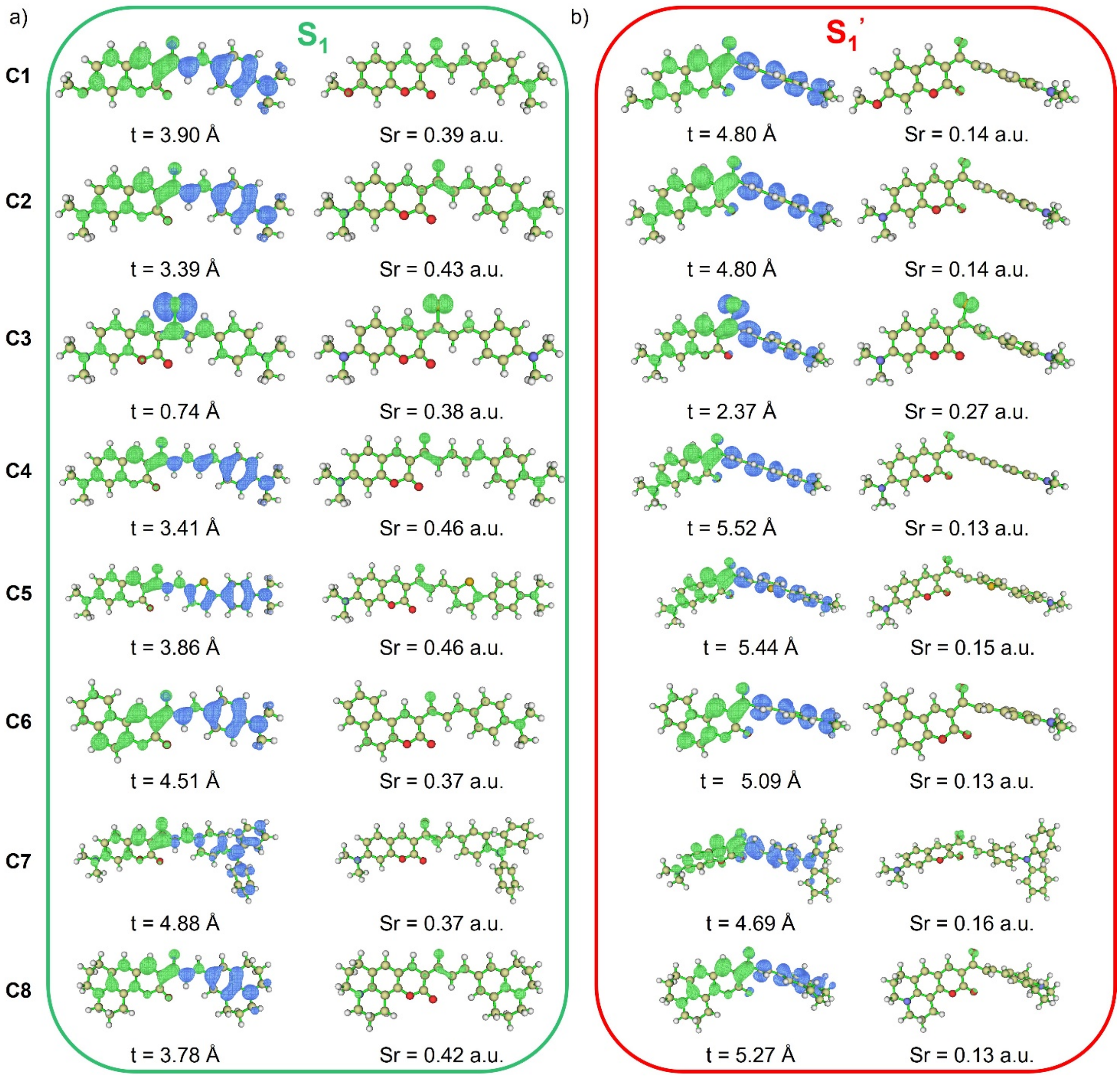

Figure S11. Hole and electron distribution and overlap of **C1-C8** at optimized (a) S_1 and (b) S_1' geometry. Blue and green isosurface represent hole and electron distributions, respectively.

Table S5. Adiabatic energy (E_{ad}) (eV) of first singlet (S_1 and S_1') and triplet (T_n) excited states of **C1-C8** PSs.

State	**C1**	**C2**	**C3**	**C4**	**C5**	**C6**	**C7**	**C8**
S_1	2.42	2.52	1.69	2.39	2.25	2.25	**2.43**	2.42
S_1'	2.20	2.35	1.67	2.27	2.24	2.05	**2.44**	2.30
T_1	1.81	1.85	1.31	1.53	1.49	1.75	1.82	1.78
T_2	2.33	2.20	1.44	2.14	2.13	2.17	2.17	2.09
T_3	2.86	2.65	1.88	2.65	2.56	2.56	2.62	2.68

Table S6. Computed excited state properties of **C1-C8** at optimized T_1 geometry. Vertical energy (E_{vt}) and wavelength emission (λ_{ems}). [a]Experimental results[1]

	E_{vt} (ev)	λ_{ems} (nm)	Transition
**C1**	1.71 (1.79)[a]	726 (690)[a]	H→L (80.6%); H→L+1 (11.9%); H-4→L (2.7%)
**C2**	1.74 (1.61)[a]	711 (770)[a]	H→L (81.2%); H→L+1 (13.2%); H-3→L+1 (13.2%)
**C3**	1.18	1043	H→L (88.0%); H-2→L (3.4%); H-3→L (3.2%); H→L+1 (2.9%)
**C4**	1.37	906	H→L (85.1%); H→L+1 (7.5%); H-2→L (3.9%)
**C5**	1.33	929	H→L (84.7%); H→L+1 (6.6%); H-2→L (5.9%)
**C6**	1.66	745	H→L (80.2%); H→L+1 (14.4%); H-4→L (2.4%)
**C7**	1.68	739	H→L (78.8%); H-2→L (9.0%); H-2→L+1 (7.8%)
**C8**	1.68	737	H→L (81.5%); H→L+1 (11.6%); H-5→L (2.2%)

Table S7. Excited state properties of **C1-C8** at optimized T_2 geometry. Vertical energy (E_{vt}) and wavelength emission (λ_{ems}). [a]Experimental results[1]

	E_{vt} (ev)	λ_{ems} (nm)	Transition
**C1**	2.20 (2.33)[a]	564 (530)[a]	H-1→L (90.4%); H-1→L+1 (2.8%); H→L+1 (2.1%)
**C2**	2.14 (2.38)[a]	578 (520)[a]	H-1→L (87.9%); H→L+1 (4.3%); H-1→L+1 (2.8%)
**C3**	1.27	974	H-1→L (97.6%)
**C4**	2.11	587	H-1→L (84.7%); H-1→L+1 (7.3%); H→L+1 (2.5%)
**C5**	2.10	589	H-1→L (83.1%); H-1→L+1 (8.8%); H→L+1 (2.1%)
**C6**	1.81	683	H-1→L (86.4%); H→L+1 (3.6%)
**C7**	2.11	587	H-1→L (84.8%); H-1→L+1 (5.5%); H→L+1 (2.8%); H-2→L (2.5%)
**C8**	2.07	600	H-1→L (86.2%); H→L+1 (6.4%); H-1→L+1 (2.9%)

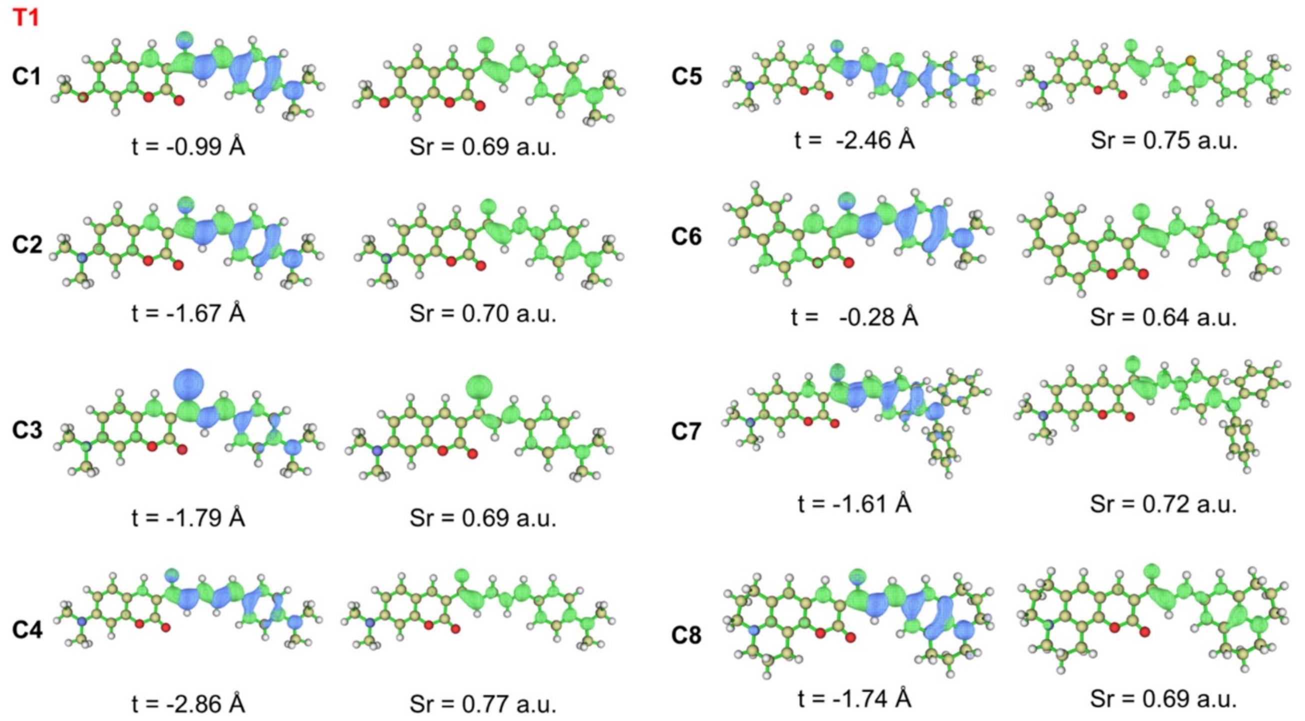

Figure S12. Hole and electron distribution and overlap of **C1-C8** at optimized T_1 geometry. Blue and green isosurface represent hole and electron distributions, respectively.

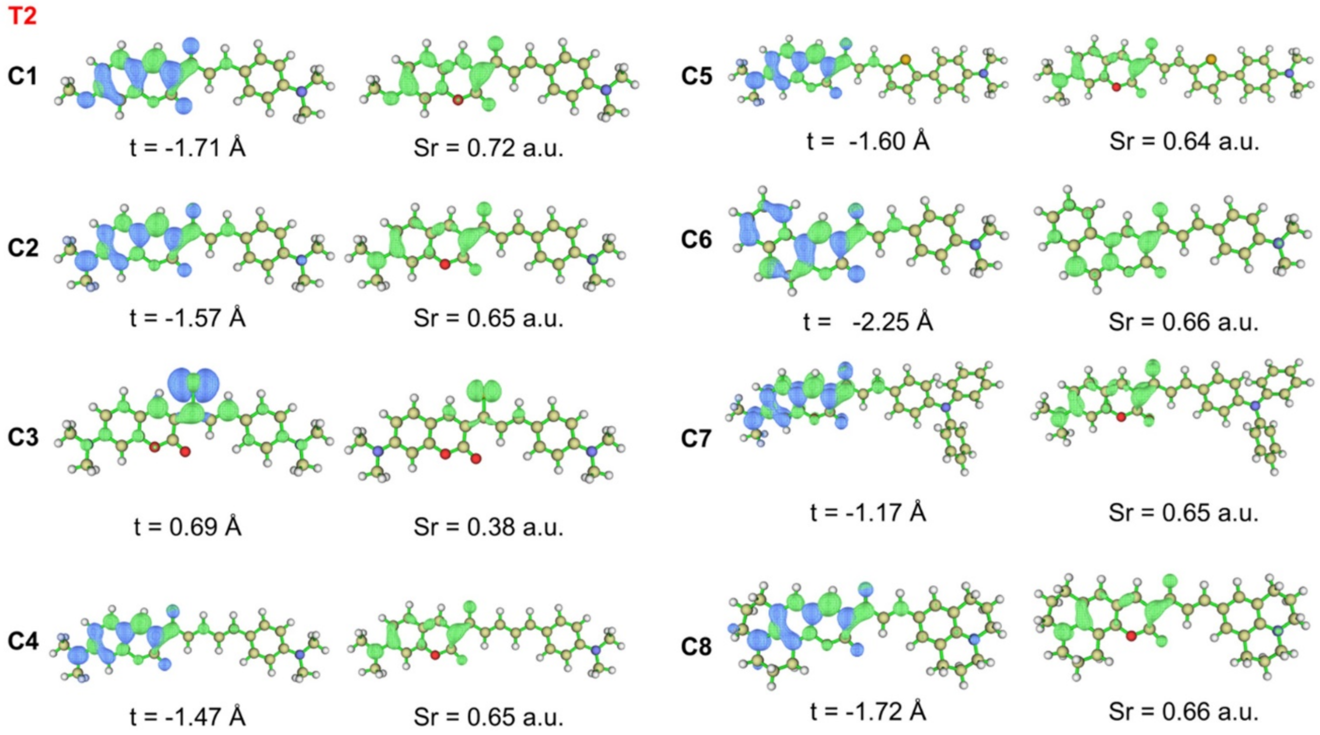

Figure S13. Hole and electron distribution and overlap of **C1-C8** at optimized T_2 geometry. Blue and green isosurface represent hole and electron distributions, respectively.

Table S8. ISC rate constant (k_{ISC}) at three triplet sublevels (M_S = 0, 1 and -1) and total along with contribution of Herzberg-Teller of $S_1 \rightarrow T_1$ transition of **C1-C8**.

	SOC (cm^{-1})	$M_S = 0$		$M_S = 1$		$M_S = -1$		**Total**
		k_{ISC} (s^{-1})	HT (%)	k_{ISC} (s^{-1})	HT (%)	k_{ISC} (s^{-1})	HT (%)	k_{ISC} (s^{-1})
**C1**	0.014	2.8×10^3	35.5	5.7×10^7	100.0	5.7×10^7	100.0	1.13×10^8
**C2**	0.010	2.0×10^3	85.5	6.2×10^7	100.0	6.2×10^7	100.0	1.24×10^8
**C3**	121.271	3.1×10^9	100.0	3.5×10^{10}	7.5	3.6×10^{10}	7.7	7.42×10^{10}
**C4**	0.000	2.1×10^2	77.0	2.6×10^6	100.0	2.6×10^6	100.0	5.26×10^6
**C5**	0.000	4.8×10^1	53.0	7.4×10^5	100.0	7.4×10^5	100.0	1.48×10^6
**C6**	0.010	2.0×10^3	17.9	2.6×10^7	100.0	2.6×10^7	100.0	5.16×10^7
**C7**	0.150	2.1×10^3	100.0	1.8×10^4	100.0	1.6×10^4	100.0	3.62×10^4
**C8**	0.024	2.7×10^3	99.3	5.1×10^5	100.0	5.1×10^5	100.0	1.02×10^6

Table S9. ISC rate constant (k_{ISC}) at three triplet sublevels (M_S = 0, 1 and -1) and total along with contribution of Herzberg-Teller of $S_1 \rightarrow T_2$ transition of **C1-C8**.

	SOC (cm^{-1})	$M_S = 0$		$M_S = 1$		$M_S = -1$		**Total**
		k_{ISC} (s^{-1})	HT (%)	k_{ISC} (s^{-1})	HT (%)	k_{ISC} (s^{-1})	HT (%)	k_{ISC} (s^{-1})
**C1**	0.010	1.9×10^2	7.2	1.0×10^7	100.0	1.0×10^7	100.0	2.00×10^7
**C2**	0.024	5.6×10^2	62.4	1.5×10^7	100.0	1.5×10^7	100.0	2.90×10^7
**C3**	0.122	5.9×10^{10}	100.0	9.4×10^{11}	4.4	9.4×10^{11}	4.4	1.94×10^{12}
**C4**	0.010	9.4×10^0	11.5	7.9×10^5	100.0	7.9×10^5	100.0	1.58×10^6
**C5**	0.140	6.5×10^1	51.6	1.2×10^4	100.0	1.2×10^4	100.0	2.40×10^4
**C6**	0.010	1.6×10^2	10.5	2.3×10^5	100.0	2.3×10^5	100.0	4.60×10^5
**C7**	0.045	2.0×10^3	100.0	2.2×10^3	98.2	2.2×10^3	98.2	6.40×10^3
**C8**	0.042	5.0×10^3	100.0	6.7×10^5	100.0	6.7×10^5	100.0	1.34×10^6

References

1. Q. Zou, Y. Fang, Y. Zhao, H. Zhao, Y. Wang, Y. Gu and F. Wu, *J. Med. Chem.*, 2013, **56**, 5288-5294.
2. J. Yin, M. Peng, Y. Ma, R. Guo and W. Lin, *Chem. Commun.*, 2018, **54**, 12093-12096.
